# Perceived influence of COVID-19 pandemic on university students' learning and mental health in Ethiopia

**DOI:** 10.1007/s11135-022-01485-8

**Published:** 2022-07-10

**Authors:** Geberew Tulu Mekonnen, Getahun Kebede Beyera, Abraham Tulu, Tigist Tolosa Roba

**Affiliations:** 1 Present Address: Faculty of Education, University of Tasmania, Launceston, TAS Australia; 2 Present Address: College of Health and Medicine, University of Tasmania, Launceston, TAS Australia; 3 College of Education, Hawassa University, Hawassa, Ethiopia; 4 College of Veterinary Medicine and Agriculture, Addis Ababa University, Addis Ababa, Ethiopia

**Keywords:** COVID-19, Pandemic, Influence, Students, Learning, Psychological and mental health

## Abstract

This study reports perceived influence of COVID-19 on students' learning and mental well-being. The data of 367 students were analysed using R. The linear regression model was fitted. A regression coefficient with 95% confidence interval (CI) was computed to identify factors associated with the perceived influence of COVID-19 on students' education and mental health. The mean ± standard deviation scores of perceived influence of COVID-19 on students' learning and communication for learning were 31.7 ± 6.7 and 21.6 ± 3.6, respectively. A similar number of students, 109 (29.7%) reported having depression and anxiety. Being a female student had a negative association with the effects of COVID-19 on learning, while being a rural resident had a strong positive association with both the effects of COVID-19 on learning and communication for learning. Similarly, being a social science student and in 2^nd^ year of study were positively associated with higher history of depression and anxiety. Residing in zonal towns, district towns, and rural settings were found to have a lower prevalence of depression and anxiety. The COVID-19 placed a serious effect on students' education and mental well-being. Thus, the Ethiopian health and higher education sectors need to provide students with basic educational resources and counselling services.

## Background of the study 

**Fig. 1:**
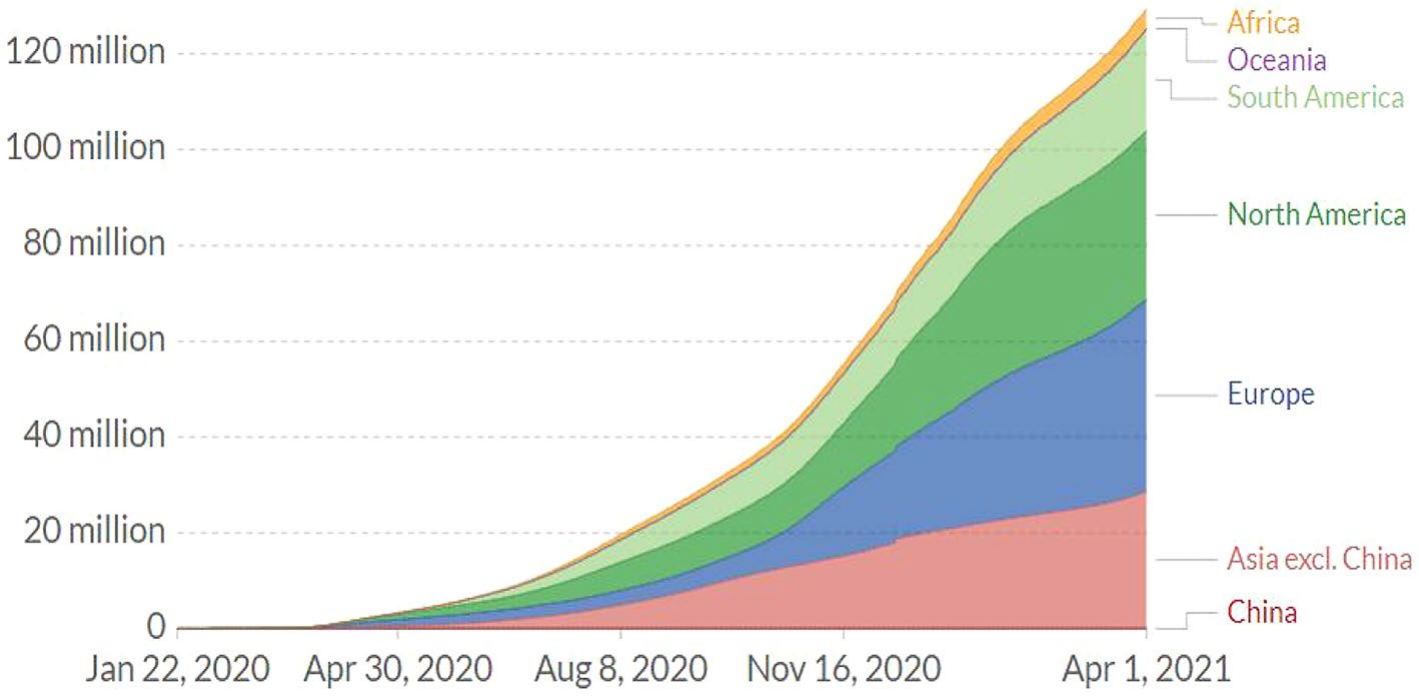
Total Confirmed COVID-19 cases. Source: European Centre for Disease Prevention and Control (2020)

Coronavirus disease 19 (COVID-19) is a respiratory illness caused by a new virus, called Severe Acute Respiratory Syndrome Coronavirus 2 (Sars-Cov-2) (Huang et al. 2020). The disease first originated in Wuhan city of China, in December 2019 and began to spread to other parts of the world in January 2020, and reached almost all countries (UNESCO 2020a). As of March 11, 2020, the World Health Organisation (WHO) also declared that the novel COVID-19 is a global pandemic. The disease is highly contagious and communicable, and the number of morbidity and mortality rates has considerably increased (Li et al. 2020). For example, Fig. 1 below demonstrates the morbidity trend from January 22, 2020 to April 01, 2021.^1^

As the COVID-19 pandemic spreads worldwide, it has created a global crisis that has had a profound influence on many countries' health, social, education and economic conditions (Abidah et al. 2020; Tewelde 2020). This crisis has brought tensions and dilemmas to several actors, including politicians, policy-makers, teachers, parents, and students (Gonzalez et al. 2020). As a result of this rapid increase in the epidemiology of COVID-19 and global crisis, many countries have activated emergency plans and established guidelines to control the spread of the disease. These involved social and physical distancing interventions, including suspending school activities, travel restrictions, and lockdown at home (Santos 2020; The World Bank 2020; WHO Emergency Committee 2020). The combination of the implementation of these public health emergency plans and the COVID-19 itself may affect the health, safety, and well-being of the students and the general population. Pfefferbaum and North (2020) argued that these effects might lead to a spectrum of emotional reactions (such as psychological distress), unhealthy behaviours (such as substance use disorder, including drinking, smoking, and chewing Khat), and non-compliance with public health directives to prevent the transmission of the disease.

As of March 18, 2020, to prevent the spread of COVID-19, an estimated 107 countries executed national school closures, affecting 862 million students, which is approximated to half the global student population (Tamrat and Teferra 2020; Viner et al. 2020). The closure of schools has also been implemented in African countries except for Eritrea, Equatorial Guinea, and Burundi (The World Bank 2020). As a result, the face-to-face teaching approach has been shifted to a virtual mode of teaching and learning untested and unprecedentedly. Student assessment is also being practised with a lot of trials and errors and uncertainties for everyone. This transition from school to home learning is not only a massive shock to students' social life and learning but also to teachers and parents (Abidah et al. 2020; The World Bank 2020). In addition, the new and unplanned learning method, where students are working on their own without proper support has placed significant challenges on students, teachers, and leaders (Tamrat and Teferra 2020). As Gonzalez et al. (2020) argued, this unprecedented situation created uncertainties for universities to manage teaching and learning, and students' learning assessments. For instance, students require a unique arrangement such as proper orientation and guidance to acclimatise themselves to this new method. However, the students were immersed in the new method without prior orientation and practical simulation (Abidah et al. 2020; Gonzalez et al. 2020; Wang et al. 2020). This may affect students' learning and performance, particularly in low-income countries.

The COVID-19 pandemic adversely destructed the education systems of both developed and developing nations. However, the situation in low-income countries is more complicated than the developed countries as the educational resources and facilities ( i.e. skilled workforce, technology, internet, and learning materials) are limited (Stewart 2016). These scarce of resources and facilities have plagued a considerable burden on Africa's higher education institutions in particular (Tamrat and Teferra 2020). As of March 26, Ethiopia suspended schools, sporting events and social gatherings when it confirmed its fifth case of the COVID-19 (Tewelde 2020). Following this, the Ministry of Science and Higher Education of the country required all the universities to change their mode of course delivery from face to face lecture to providing lectures via e-mail, webpage and other online platforms (Addis Ababa University 2020). However, teaching online is not that easy in a region where only 19% of the population has access to the internet (World Bank 2017), poor connectivity, high cost, and frequent power interruption (Tamrat and Teferra 2020). This limited availability and accessibility of the internet may affect not only students' learning, but also their psychological and mental well-being. Nevertheless, the influence of this outbreak on students' learning, psychological and mental health is neglected. This study is, therefore, aimed to answer the following research questions:

1. What are theperceived influences of COVID-19 pandemic on students' learning, and mental and psychological well-being?2. What factors are associated with perceived influences of COVID-19 pandemic on students' learning, and mental and psychological well-being?

### Perceived influence of COVID-19 on students' education and well-being

Although the cumulative impact of COVID-19 on university students' education and mental health is unclear, the pandemic is anticipated to cause considerable consequences (Saha et al. 2020) in both education and health systems. For example, recent studies (Browning et al. 2021; Chaturvedi et al. 2021; Ihm et al. 2021; Islam et al. 2020; Osorio-Saez et al. 2021; Sundarasen et al. 2020) documented that a sudden move to online learning, social distancing and reduced interaction with teachers and peers, closure of campuses, social isolation, fear of infection, and loss of family members are among the consequences of COVID-19 on higher education students' education and well-being. Another recent study was undertaken on Chinese medical college students (Cao et al. 2020) and also revealed that a higher level of anxiety was associated with COVID-19. Furthermore, the data from international students demonstrated a rise in worries students about their education and their well-being and that of their families in case they return home (Zhai and Du 2020). As a result of the change in the mode of course delivery and uncertainty of the continuity of the online teaching of university education, technological issues associated with online course delivery, being away from home, social alienation, and financial challenges, the number of students experienced increased stress, anxiety, and depressive symptomatology (Browning et al. 2021; Cao et al. 2020; Chaturvedi et al. 2021; Zhai and Du 2020). However, there is a paucity of data about perceived influence of the COVID-19 pandemic on higher education students' education and well-being in Ethiopia (Tamrat 2021; Zhai and Du 2020). This is study, therefore, timely to derive the data about perceived influence of COVID-19 on higher education students' learning, and mental and psychological well-being in Ethiopia.

### Definition of key terms

Student learning refers to students' learning engagement or understanding in a particular program or course in various ways, including planning, time management, discussion, assignment completion, and using different learning space and materials (e.g., Library resources, lecture notes, or handouts).

Communication for learning: The use of various means (email, face to face discussion, social media etc.) to communicate with students, peers, and teachers, is referred to as communication for learning.

## Methods

### Study design and setting

A population-based cross-sectional study was conducted in May 2020 among higher education students in Ethiopia.

### Study sample and sampling procedure

The sample size for the study was calculated with the expected frequency of depression (p = 50%), 95% level of confidence, 5% margin of error. An anticipated 10% non-response rate was also considered to obtain the final sample size of 422 students.

A multi stage sampling technique was used to select the sample students. Firstly, the over 40 universities in the country stratified into four generations (1^st^ generation, established betwwen 1950–2004/, 2^nd^ generation established betwwen 2005–2010,3^rd^ generation established betwwen 2011–2016, and 4^th^ generation established betwwen 2017–2019) based on their year of establishment. Secondly, considering similarities within and differences between each generation universities in their resources, facilities, academic programs, and number of students (which differ between each generation universities but almost similar within each generation), one university was selected randomly from each generation.. Thirdly, the lists of students in each program (sampling frame) were obtained in communication with the heads of academic units of the sampling universities. Finally, a simple random sampling technique with proportional allocation was then applied using the OpenEpi Random Number generator to select the study participants.

#### Inclusion criteria

All regular university students were included in the study.

### Data collection and instrument

The data were collected using an online survey questionnaire with the assistance of the heads of academic units, who disseminated the survey questionnaire to the selected students via their e-mail addresses. The questionnaire was designed in English language. This is based on the premise that English is used as the medium of instruction and that the students' education level is necessary to understand and respond to the questions. Questions including socio-demographic information, self-perceived influence of COVID-19 on students' learning, and psychological and mental health were developed after reviewing related literature intensively (Auerbach et al. 2016; Browning et al. 2021; Cao et al. 2020; Chaturvedi et al. 2021; Zigmond and Snaith 1983). Theperceivedinfluences of COVID-19 on students' education were assessed by 13 items with a 5-point Likert scale ranging from '1' (strongly disagree) to '5' (strongly agree). The first eight items measured the perceived influence of the COVID-19 on students' learning, and the latter five items measured the perceived influence the pandemic on communication for learning. Psychological and mental health status was measured by 12 items (each with a 5-point Likert scale ranging from '1' (never) to '5' (always) assessing depression and anxiety levels, each with six items i.e. the first six items measuring depression while the last six items measuring anxirty). The internal consistency of each scale, forming the online survey questionnaire was measured using *Cronbach's xalpha (α)*. Accordingly, the scale to measure perceived influence of COVID-19 on students' learning (α = 0.845), communication for learning (α = 0.801), depression (α = 0.891), and anxiety (α = 0.889), were shown to have acceptable levels of internal consistency reliability when applied to the same study population.

### Definitions

To describe the psychological and mental health status of students as a proportion of students having depression and anxiety, the following concepts were used. As conceptualised in the literature (Zigmond and Snaith 1983), individuals who scored ≥ 20 out of a total possible 30 points on a scale assessing depression level, were defined as having depression, and those with a score of ≤ 13 and 14–19, respectively were defined as borderline cases and normal (have no depression). The same procedure was applied to define individuals' anxiety levels.

### Statistical analyses

The data were analysed using R. Statistical measures, such as percentages, means, standard deviations, and confidence intervals (CIs) and computed for essential variables. The linear regression model was fitted, and both unadjusted and adjusted beta (β) or regression coefficients with 95% CIs were calculated to identify factors associated with the influences of COVID-19 on students' education, and their psychological and mental health. The significance level was considered at the p-value ≤ 0.05.

*Ethical approval.*

Ethical approval for this study was obtained from one of the universities in Ethiopia. Informed consent was also obtained from all the students who participated in the study. Participation in the study was voluntary, and confidentiality was maintained at all times.

## Results

### Socio-demographic characteristics of the study participants

A total of 367 students completed the online survey questions, which made a response rate of 87%. Of the total participants, about one-third were male. The participants' ages range from19-50 years with a median (interquartile range [IQR]) of 28 (23–43) years (Table 1).

**Table 1:** Socio-demographic Characteristics of the Study Participants

Variable		Frequency	%
Sex	Male	274	74.7
	Female	93	25.3
Age in years, median (IQR)		28 (23–34)	-
Residential area	Addis Ababa	135	36.8
	Regional city	66	18.0
	Zonal town	105	28.6
	District town	48	13.1
	Rural	13	3.5
Study stream	Natural science	251	68.4
	Social science	116	31.6
Study program	Undergraduate	220	59.9
	Postgraduate	147	40.1
Study year	1^st^ year	136	37.1
	2^nd^ year	85	23.2
	3^rd^ year	63	17.2
	4^th^ year and above	83	22.6

IQR: Interquartile range

### The perceived influence of COVID-19 on students' education and their psychological and mental well-being

The mean ± standard deviation (SD) scores of perceived influence of COVID-19 on students' learning and communication for learning were 31.7 ± 6.7 and 21.6 ± 3.6, respectively. In addition, the mean score of depression ± SD was 16.3 ± 5.9, while the mean score of anxiety + SD was 15.9 + 6.2. Specifically, when the concepts defined above in the methods are applied, a total of 109 (29.7%, 95% CI 25.3–34.6) and another 109 (29.7%, 95% CI 25.1–34.3) students reported having depression and anxiety, respectively.

### Factors associated with the influences of COVID-19 on students' education

**Fig. 2:**
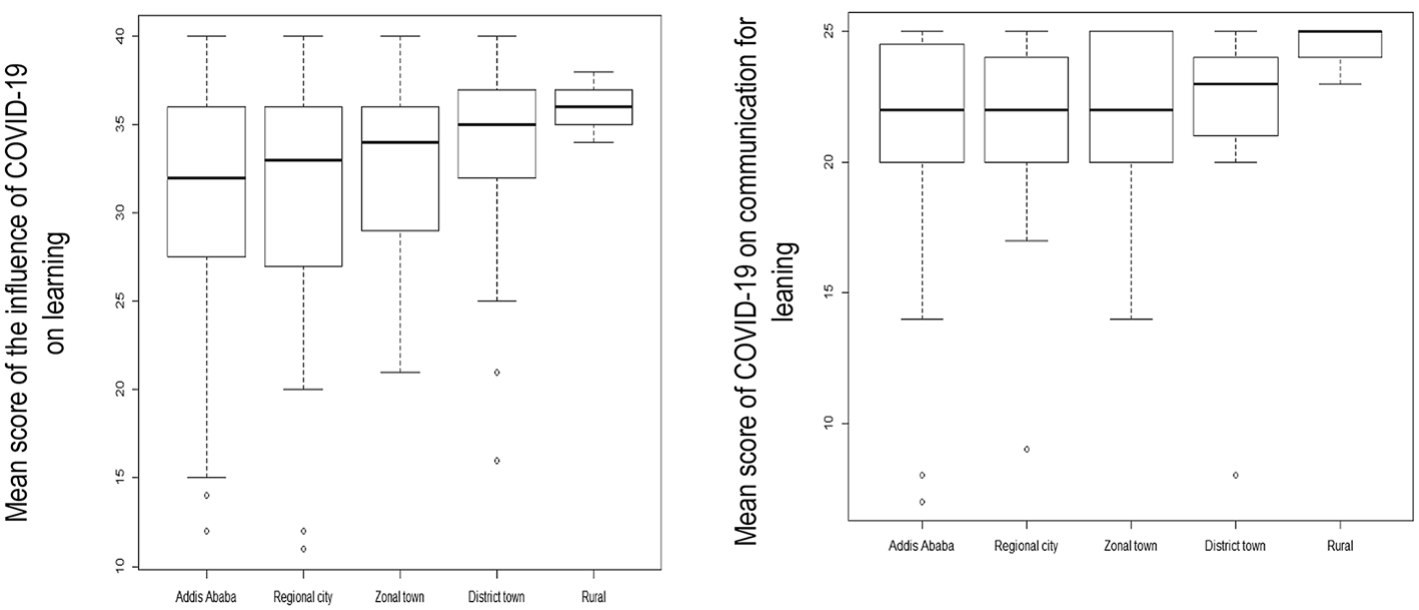
Boxplots Showing the Association between Residential Settings and the Influence of COVID-19 on Students’ Education

The linear regression analysis showed that four factors, students' sex, residential setting, study program, and year of study, were associated with the influences of COVID-19 on their learning. When adjusted for students’ residential settings, females were found to have 2.74 and 1.32 lower mean scores of perceived influence of COVID-19 on their learning (β = -2.74, 95% CI -4.33 to -1.16) and communication for learning (β = -1.32, 95% CI -2.19 to -0.46), respectively compared to male students. As demonstrated in Fig. 2 and the unadjusted regression coefficients in Table 2, perceived influence of the pandemic on students' learning was different across residential settings except in regional cities. However, when adjusted for sex, the difference was statistically significant only between rural students and students living in the capital city, Addis Ababa. Students living in rural settings reported a higher mean score of the perceived influence of the pandemic on their learning than those living in Addis Ababa (β = 4.82, 95% CI 1.09–8.54). Similarly, rural students had a 2.80 higher mean score of perceived influence of COVID-19 on their communication for learning (β = 2.80, 95% CI 0.78–4.83) (Table 2).

**Table 2:** Factors Associated with the Perceived Influences of Covid-19 on Students' Education

Variable	Association with perceived influence of COVID-19 on learning				Association with perceived influence of COVID-19 on communication for learning			
	β (95% CI)	*p-value*	β^‡^ (95% CI)	*p-value*	β (95% CI)	*p-value*	β^‡^ (95% CI)	*p-value*
Sex (Male^†^)								
Female	-3.22 (-4.76 to -1.67)	** < *0.001***	-2.74 (-4.33 to − 1.16)	** < *0.001***	-1.42 (-2.25 to − 0.58)	** < *0.001***	-1.32 (-2.19 to − 0.46)	***0.003***
Age	-0.03 (-0.13 to 0.06)	*0.511*	-0.05 (-0.15 to 0.05)	*0.330*	0.01 (-0.04 to 0.06)	*0.760*	0.01 (-0.05 to 0.06)	*0.782*
Residential place (Addis Ababa^†^)								
Regional city	0.38 (-1.56 to 2.33)	*0.700*	-0.07 (-2.01 to 1.86)	*0.941*	0.08 (-0.97 to 1.14)	*0.876*	-0.14 (-1.19 to 0.92)	*0.800*
Zonal town	2.19 (0.50–3.87)	***0.011***	1.61 (-0.09 to 3.30)	*0.063*	0.16 (-0.75 to 1.08)	*0.724*	-0.12 (-1.04 to 0.81)	*0.804*
District town	2.61 (0.43–4.79)	***0.019***	1.76 (-0.44 to 3.96)	*0.116*	0.87 (-0.31 to 2.05)	*0.149*	0.46 (-0.74 to 1.65)	*0.453*
Rural	5.47 (1.71–9.23)	***0.004***	4.82 (1.09–8.54)	***0.011***	3.12 (1.08–5.16)	***0.003***	2.80 (0.78–4.83)	***0.007***
Study stream (Natural science^†^)								
Social science	-1.27 (-2.75 -0.20)	*0.090*	-0.12 (-1.69 to 1.45)	*0.882*	-0.97 (-1.76 to − 0.18)	***0.017***	-0.73 (-1.58 to 0.12)	*0.093*
Study program (Undergraduate^†^)								
Postgraduate	-0.42 (-1.82 to 0.98)	*0.555*	-0.04 (-1.51 to 1.43)	*0.959*	-1.02 (-1.77 to − 0.27)	***0.008***	-1.12 (-1.91 to − 0.33)	***0.005***
Study year (1^st^ year^†^)								
2^nd^ year	1.43 (-0.39 to 3.24)	*0.123*	1.94 (0.14–3.74)	***0.034***	1.24 (0.28–2.21)	*0.012*	1.27 (0.30–2.24)	***0.010***
3^rd^ year	1.18 (-0.82 to 3.18)	*0.247*	1.54 (-0.45 to 3.52)	*0.129*	-0.75 (-1.81 to 0.32)	*0.168*	-0.80 (-1.87 to 0.26)	*0.140*
4^th^ year and above	1.66 (-0.17 -3.49)	*0.075*	1.97 (0.11 to 3.83)	***0.038***	0.44 (-0.53 to 1.41)	*0.376*	0.31 (-0.69 to 1.31)	*0.540*

β: Regression coefficient (unadjusted); ^†^: Reference category; ^‡^: adjusted for sex and residential place

Although perceived influence of COVID-19 on students' learning was not different between study programs (β = -0.04, 95% CI -1.51—1.43), perceived influence of the pandemic on communication for learning was lower in postgraduate students than undergraduate students (β = -1.12, 95% CI -1.91 to − 0.33). Perceived influence of the pandemic on students' learning was higher among the second-year (β = 1.94, 95% CI 0.14—3.74), and fourth-year and above students (β = 1.97, 95% CI 0.11—3.83) when compared with first-year students. Second-year students had a further higher influence of the pandemic on their communication for learning than first-year students (β = 1.27, 95% CI 0.30—2.24) (Table 3).

**Table 3:** Factors Associated with Psychological and Mental Health Effects

Variable	Association with depression				Association with anxiety			
	β (95% CI)	*p-value*	β^*§*^ (95% CI)	*p-value*	β (95% CI)	*p-value*	β^§^ (95% CI)	*p-value*
Sex (Male^†^)								
Female	-1.54 (-2.92 to − 0.16)	***0.029***	-0.29 (-1.58 to 0.99)	*0.653*	-0.22 (-1.69 to 1.24)	*0.764*	0.86 (-0.55 to 2.27)	*0.232*
Age	-0.02 (-0.10 to 0.07)	*0.661*	-0.01 (-0.09 to 0.07)	*0.802*	-0.06 (-0.15 to 0.03)	*0.174*	-0.05 (-0.14 to 0.03)	*0.205*
Residential place (Addis Ababa^†^)								
Regional city	-1.18 (-2.90 to 0.54)	*0.180*	-1.32 (-2.86 to 0.21)	*0.091*	-1.28 (-3.12 to 0.55)	*0.171*	-1.40 (-3.11 to 0.30)	*0.107*
Zonal town	-2.12 (-3.61 to − 0.62)	***0.005***	-2.93 (-4.27 to − 1.58)	** < *0.001***	-1.89 (-3.48 to − 0.30)	***0.020***	-2.58 (-4.07 to − 1.08)	** < *0.001***
District town	-0.86 (-2.79 to 1.07)	*0.381*	-1.90 (-3.63 to − 0.17)	***0.031***	-1.01 (-3.06 to 1.04)	*0.333*	-1.90 (-3.83 to 0.02)	*0.052*
Rural	1.27 (-2.06 to 4.60)	*0.454*	-1.06 (-4.08 to 1.95)	*0.488*	-1.51 (-5.05 to 2.02)	*0.401*	-3.53 (-6.87 to − 0.18)	***0.039***
Study stream (Natural science^†^)								
Social science	2.10 (0.81–3.38)	** < *0.001***	2.70 (1.55–3.85)	** < *0.001***	1.95 (0.59–3.31)	***0.005***	2.45 (1.17–3.73)	** < *0.001***
Study program (Undergraduate^†^)								
Postgraduate	-0.37 (-1.60 to 0.87)	*0.559*	-0.07 (-1.20 to 1.05)	*0.897*	0.52 (-0.78 to 1.82)	*0.435*	0.78 (-0.46 to 2.02)	*0.216*
Study year (1^st^ year^†^)								
2^nd^ year	2.79 (1.22–4.37)	** < *0.001***	2.18 (0.74–3.62)	***0.003***	3.05 (1.39–4.72)	** < *0.001***	2.54 (0.96–4.12)	***0.002***
3^rd^ year	1.05 (-0.69 to 2.79)	*0.235*	0.75 (-0.84 to 2.35)	*0.354*	1.81 (-0.02 to 3.65)	*0.052*	1.57 (-0.18 to 3.33)	*0.078*
4^th^ year and above	0.53 (-1.06 to 2.12)	*0.511*	-0.07 (-1.51 to 1.38)	*0.927*	0.40 (-1.28 to 2.07)	*0.640*	0.09 (-1.68 to 1.49)	*0.908*
Influence of COVID-19 on students’ learning	0.38 (0.30–0.46)	** < *0.001***	0.32 (0.21–0.43)	** < *0.001***	0.31 (0.22–0.40)	** < *0.001***	0.27 (0.15–0.39)	** < *0.001***
Influence of COVID-19 on students’ communication for learning	0.55 (0.40–0.71)	** < *0.001***	0.15 (-0.05 to 0.36)	*0.138*	0.46 (0.29–0.63)	** < *0.001***	0.13 (-0.10 to 0.35)	*0.266*

β: Regression coefficient (unadjusted); ^†^: Reference category; ^§^: adjusted for perceived influences of COVID-19 on leaning and communication for learning

### Factors associated with psychological and mental well-being of the students

The students' mean scores of depression and anxiety levels were statistically associated with their residence, study stream, year of study, and mean score of perceived influence of COVID-19 on learning. When compared with those living in Addis Ababa, students living in the zonal towns had lower depression (β = -2.93, 95% CI -4.27 to − 1.58) and anxiety (β = -2.58, 95% CI -4.07 to − 1.08) levels; similarly, those living in the district towns and rural settings had lower mean scores of depression (β = -1.90, 95% CI -3.63 to − 0.17) and anxiety (β = -3.53, 95% CI -6.87 to − 0.18) levels. Social science students had 2.70 and 4.45 higher levels of depression (β = 2.70, 95% CI 1.55—3.85) and anxiety (β = 2.45, 95% CI 1.17—3.73) than natural science students, respectively. The levels of both depression (β = 2.18, 95% CI 0.74—3.62) and anxiety (β = 2.54, 95% CI 0.96—4.12) were also higher in second-year students by 2.18 and 2.54 than first-year students, consecutively. There was a statistically significant association between perceived influence of COVID-19 on students' learning and psychological and mental well-being. A unit increase in the mean score of perceived influence of COVID-19 on students' leaning was associated with a 0.32 increase in students' mean score of depression level(β = 0.32, 95% CI 0.21—0.43). Similarly, a unit increase in the mean score of perceived influence of COVID-19 on students' leaning was associated with a 0.27 increase in their mean score of anxiety level (β = 0.27, 95% CI 0.15—0.39) (see Table 3).

## Discussion

This study investigated perceived influence of the currently emerged COVID-19 pandemic on students' education, psychological and mental well-being in Ethiopian higher education institutions. From the maximum possible points, 40 (for perceived influence of COVID-19 on students' learning) and 25 (for perceived influence of COVID-19 on students' communication for learning), the mean + standard deviation (SD) scores were 33 + 6.7 and 23 + 3.6, respectively. These figures indicate that COVID-19 pandemic is placing a considerable influence on students' education. The results of the study also revealed that the students' sex, residential setting, study program, and year of study, were associated with perceived influence of COVID-19 on their education. The influence of the pandemic on students learning and communication for learning was lower in females than male students. This result is consistent with Dong et al. (2020), who found that more boys than girls were affected by the COVID-19 pandemic. The pandemic also affected students' learning differently across their residential settings. Rural students' learning and communication for learning were more affected by the pandemic than those who reside in Addis Ababa. This could be attributed to the fact that students who live in the rural are more likely to have poor or no internet access as they cannot afford the cost of a laptop/computer or internet connection during the pandemic (UNESCO 2020b). Tamrat and Teferra (2020) further argued that learning and communication are not that easy for students from rural areas in Ethiopia, where only 19% of the population has access to the internet, poor connectivity, high costs, and frequent power interruptions. Similar problems have been exhibited across different countries, although variations have been observed with Africa having the lowest (39.3%) and North America having the highest (94.6%) internet penetration. This idea is further supported by Vegas (2020), who reported that in sub-Saharan Africa, only 11% of countries rely solely on online learning. This limited access to the internet apparently exacerbates students' learning in rural areas, exclusively during the pandemic.

The findings of this study further disclosed that there was a variation in perceived influence of COVID-19 on students' education across study years. Perceived influence of the pandemic on students' education was higher among the second-year, and fourth-year and above than first-year students. This could be attributed to a shortage of time to complete their learning tasks as most of the second-year (postgraduate), and fourth-year and above students are at the final stage of their study. On the other hand, the perceived influence of the pandemic on postgraduate students' communication for learning was lower compared to undergraduate students. This could be ascribed to the fact that most postgraduate students are economically and socially in a better position when seen the light of undergraduate students securing the internet and other learning materials and interacting with instructors. UNESCO (2020a, b) also noted that students' and their families' socio-economic conditions are aggravating factors for the digital divide.

The pandemic affects not only the students' education, but also their psychological and mental well-being. A previous study undertaken before the emergence of the pandemic showed that 20.3% of college students had one or more diagnosable mental health issues globally (Auerbach et al. 2016). Perceived influence of COVID-19 on students' education may raise this figure and exacerbate students' psychological and mental health disorders (Zhai and Du 2020), which is of particular concern in low-income countries like Ethiopia due to limited access to basic educational resources, such as internet. In complement with this idea,, 29.7% of the students reported having depression in this study, and a similar number (29.7%) of the students showed having anxiety. These figures are lower than the findings of the study conducted among high school students in China, where 43.7% and 37.4%, respectively showed to have depressive symptoms and anxiety during the novel COVID-19 outbreak (Zhou et al. 2020). This difference may be linked to the difference in the study population. The study population for this study were university students, whilst that of the Chinese study were high school students. The sociocultural difference between the two countries may also significantly contribute to the observed variation in the prevalence of depression and anxiety across the two studies.

When compared with those living in Addis Ababa, students residing in the zonal towns were found to have lower levels of depression and anxiety. In addition, the levels of depression and anxiety, respectively, were lower in those living in the district towns and rural settings than students living in Addis Ababa. This means that participants residing in the capital were at high risk of having psychological and mental health problems. These findings are in accordance with a recent study showing that living in the worst-hit areas by the COVID-19 was a significant predictor of depression (Tang et al. 2020). In Ethiopia, Addis Ababa is the epicentre of the pandemic or the highest-hit area. Of the total 221,544 confirmed cases in the country as of April 08, 2021, 123,660 cases and 2,147 (70.2%) of the 3,058 deaths occurred in Addis Ababa (Addis Ababa City Administration Health Bureau Public Heath Emergency Operation Center 2021). Therefore, the higher levels of depression and anxiety reported in this study could be associated with this fact. Evidence further demonstrates that in the most affected settings by the pandemic, universities encounter the prospect of missing an entire semester or even beyond (de Oliveira Araújo et al. 2020). In the Ethiopian context, students from rural, district, and zonal areas are more likely to be involved in helping their parents compared to those in the urban (Tafere 2014). Since those areas are less affected by COVID-19, social interaction is also likely to be higher, and which could be one of the reasons why students residing in those areas were found to have lower depression and anxiety. When it comes to the impact of COVID-19 on students' learning and communication for learning, those who live in rural, district, and zonal areas are more likely to be affected than those who live in urban because of lack of internet access and learning resources. Drawing on this idea, Belay (2020) argued that due to COVID-19 outbreak, rural students are in a disadvantaged position in their education compared to urban students due to poor internet connectivity and a shortage of electronic devices s (e.g., mobile phones, laptops, etc.). Thus, the longer a university is closed, the more students in rural, district, and zonal areas are preoccupied with supporting their parents with various activities such as farming, livestock herding, and business. This may cause them to become disengaged from their studies. This study showed that students in their second year of study had significantly higher mean scores of depression anxiety levels than first-year students. Tang et al. (2020) also showed that being in the final year of university study was a risk factor for developing psychological distress. The higher levels of depression and anxiety among second-year students may, therefore, be due to the overrepresentation of second-year students by postgraduate students, who are a graduating class. Moreover, Tang et al. (2020) argued that the uncertainties about the possible influences of the pandemic may cause graduating class students to worry about their future, including their graduation, finding a job and/or enrolment in further study.

There is evidence that students' academic discipline is associated with their history of reporting depression and anxiety (Posselt and Lipson 2016). In the current study, students studying social science were found to have significantly higher levels of depression and anxiety than those studying natural science. One of the possible explanations for this difference could be linked to students' levels of knowledge and skills in utilising electronic devices (such as computer/laptop) and internet to browse educational resources from various relevant websites. In Ethiopia, while natural science students begin studying the subject of computer science in grade 11, social science students subject to this course after higher education enrolment. Thus, although the study stream had no significant association with the influence of COVID-19 on learning and communication for learning in this study, the effect it has on higher education students may be broader than having association with depression and anxiety, and it should not be undermined, and further investigation could be important.

The study among university students in Bangladesh (Islam et al. 2020) indicated thatnearly two-thirds of university students were depressed during the lockdown for fear of falling academically behind their peers. Islam et al. (2020) further argued that online learning had adverse effects on students' psychological and mental well-being because the online classes could not fulfill their requirements during the lockdown. In agreement with this argument, a higher mean score of perceived influence of the COVID-19 pandemic on students' learning was associated with higher depression and anxiety levels in this study. Malee and Arnhold (2020) further claimed that quite a lot of formally enrolled students in tertiary education were affected by the pandemic.

### Strengths and limitations

One of the strengths of this study is that it considered all students in each generation universities in the country and provided a piece of firsthand evidence to education and public health policy-makers and researchers. However, the 13% non-response rate created a discrepancy with the 10% non-response rate factored in the sample size determination. Those students in rural areas with very limited access to internet services and a higher influence of the COVID-19 may not be able to participate and underrepresented in the study, underestimating the reported influence of the pandemic on students' learning and mental health. Considering the subjective and self-report nature of the survey questions, students might overestimate the impact of the COVID-19 on their learning and mental wellbeing. Bowman and Seifert (2011) also noted that students' self-reporting of their learning could be exaggerated and therefore, future studies with triangulation tools are important.

## Conclusions

The findings of this study are evident that the COVID-19 pandemic placed a serious influence on university students' education, and adversely affected their psychological and mental well-being as well. Students' sex, residential setting, study program, and year of study, were independently associated with perceived influences of COVID-19 on their education. In addition, residential setting, study stream, year of study, and mean score of perceived influence of COVID-19 on students' learning, were statistically associated with depression and anxiety levels. The Ethiopian higher education sector needs to make the necessary arrangements for university students to access free internet services for online learning during the pandemic. Students in rural settings also need special attention. Provision of psychological interventions that reduce the mental health influence of the pandemic among the students would also be important, with particular consideration to those residing in the Addis Ababa (the worst-hit area by the pandemic) and graduating class students. A collaborative work between the Ethiopian health and higher education sectors would also equally be essential to achieve the desired results in this regard. Similar studies investigating perceived influence of COVID-19 on students' learning and well-being could be undertaken in the context of other developing countries to establish a more comprehensive picture of COVID-19's impact on higher education.

## Appendix

### Survey instrument

#### Perceived influenceof COVID-19 on higher education studentsin Ethiopia

Dear student,

We are academicians. We want to collect data about “Perceived infulence of COVID-19 on higher education students in Ethiopia” using online data collection platform. Filling this online survey will take about 20 min. The questionnaire is anonymous; you are not asked to provide your name or contact information on the questionnaire. The data from this questionnaire will be kept confidential and used only for this study. The reliability and validity of this study are entirely dependent on the quality of your response. You are, therefore, kindly requested to give your frank responses.

Thank you for your participation.

## Part 1: socio-demographic information

1. Sex:
2. Female
3. Male

2. Age in years
3. Field of study

1. Social science
2. Natural science

4. Program of the study

1. Undergraduate
2. Postgraduate

5. year of study

1. 1.^st^ year
2. 2.^nd^ year
3. 3.^rd^ year
4. 4.^th^ year
5. 5.^th^ year
6. 6.^th^ year

6. Current residential place

1. Addis Ababa
2. Regional city
3. Zonal town
4. District town
5. Rural

## Part 2: Perceived influence of COVID-19 on Learning

Please make a tick mark “√” against the items indicating the extent to which you agree or disagree with the following statements. Each item in the table applies to your response using the rating scales (ranging from 1 to 5):

Strongly disagree = 1; Disagree = 2; Neutral = 3; Agree = 4; Strongly agree = 5

**Table:**

1. I feel COVID-19 caused a delay in courses coverage	1	2	3	4	5
2. The COVID-19 affected my understanding of the course content					
3. The COVID-19 affected my peer group discussion					
4. The COVID-19 affected to submit the assignment before/on the due date					
5. The COVID-19 affected my academic performance					
6. The COVID-19 affected my communication on academic matters with my instructors					
7. The COVID-19 affected my communication with my classmates					
8. The COVID-19 affected my internet accessibility to attend online learning					
9. The COVID-19 affected my access to learning materials (e.g. Library resources, lecture notes or handouts)					
10. The Covid-19 affected my learning plan					
11. The COVID-19 affected my study time					
12. The Covid-19 affected my movement for learning					
13. The Covid-19 affected the support I get from instructors					

## Part 3: The psychological and mental consequence caused by the impact of CPVID-19

Please make a tick mark “√” against the items indicating how often have you been feeling the following problems. Each item in the table applies to your response using the rating scales (ranging from 1 to 5):

Never = 1; rarely = 2; sometimes = 3; most of the time = 4; Always = 5.

**Table:**

Because of the impact of COVID-19 on my learning,						
14. I have been feeling little interest in doing things	1	2	3	4		5
15. I have been feeling down						
16. I have been feeling depressed						
17. I have been feeling hopeless						
18. I have been feeling bad about myself						
19. I have been feeling trouble concentrating						
20. I have been feeling nervous						
21. I have been feeling worried						
22. I have been feeling trouble relaxing						
23. I have been feeling of being restless						
24. I have been feeling afraid						
25. I have been feeling helpless						

## Footnotes

1 At the time this study started, vaccinations were not available anywhere.
